# A simple noninvasive model to predict significant fibrosis in children with chronic hepatitis B

**DOI:** 10.1097/MD.0000000000026462

**Published:** 2021-06-25

**Authors:** Kang-Ling Zhang, Xiu-Qi Chen, Zi-Li Lv, Qing Tang, Qing-Wen Shan

**Affiliations:** a Department Of Pediatrics; b Department Of Pathology, The First Affiliated Hospital Of Guangxi Medical University, Nanning; c Child Health Section, Maternal and Child Health Hospital of Hubei Province, Tongji Medical College, Huazhong University of Science and Technology, Wuhan, China.

**Keywords:** children, diagnostic model, fibrosis, hepatitis B virus, liver biopsy

## Abstract

To develop a noninvasive model to predict significant fibrosis in children with chronic hepatitis B (CHB).

A total of 116 CHB pediatric patients who underwent liver biopsy were included in the study. Liver histology, which is the gold standard for assessing fibrosis, was performed. Blood routine examination, coagulation function, liver biochemistry, viral serology, and viral load were analyzed. Receiver operating characteristic curve analysis was used to analyze the sensitivity and specificity of all possible cut-off values.

Based on the correlation and difference analyses, 7 available clinical parameters (total bile acid, gamma-glutamyl transpeptidase [GGT], aspartate transaminase, direct bilirubin to total bilirubin ratio, alanine aminotransferase, prealbumin [PA], and cholinesterase) were included in the modeling analysis. A model to predict significant liver fibrosis was derived using the 2 best parameters (PA and GGT). The original model was Y=−1.803 ln PA(mg/L)+0.769lnGGT(U/L)+6.436. After the mathematical calculation, the G index=600 × GGT/PA^2^ predicted significant fibrosis, with an area under the receiving operating characteristics (AUROC) curve of 0.733, 95% confidence interval (0.643–0.811). The AUROC of the G index (0.733) was higher than that of aminotransferase to platelet ratio index (APRI) (0.680) and Fibrosis index based on 4 factors (FIB-4) (0.601) in predicting significant fibrosis in children with CHB. If the values of the G index were outside the range of 0.28 to 1.16, 52% of children with CHB could avoid liver biopsy, with an overall accuracy of 75%.

The G index can predict and exclude significant fibrosis in children with CHB, and it may reduce the need for liver biopsy in children with CHB.

## 1 Introduction

The widespread infection of the hepatitis B virus (HBV) is a global public health problem.^[1]^ HBV infection is the most common cause of cirrhosis and hepatocellular carcinoma in China.^[2]^ The misdiagnosis or non-treatment of children with chronic hepatitis B (CHB) is an important factor that leads to the development of end-stage liver disease in adulthood.^[3]^ Therefore, it is important to obtain antiviral treatment in a timely and accurate manner to prevent children with CHB from developing end-stage liver disease.^[4,5]^

Children with CHB lack typical clinical signs and symptoms, most of which are found during a health check-up. There may be severe histopathological changes in the liver in pediatric patients due to their immune tolerance, although their alanine aminotransferase (ALT) is normal.^[6]^ For children with normal liver biochemistry, the indications for antiviral therapy need to rely on the liver biopsy (S ≥2 or G ≥2). However, children have a higher risk and a lower success rate in liver biopsy than adults. Moreover, it is difficult to dynamically assess fibrosis through liver biopsy.

Although many noninvasive models have been established, the model data were from adults with CHB or chronic hepatitis C. The classical models for adults, such as aminotransferase to platelet ratio index (APRI)^[7]^ and Fibrosis index based on 4 factors (FIB-4)^[8]^ have not been verified in children, and there are only a few reports about noninvasive models for children with CHB. Thus, this study developed a simple, noninvasive model to predict fibrosis in pediatric patients.

## 2 Methods

### 2.1 Patients

This retrospective study included 116 patients with CHB (age <15 years) who underwent liver biopsy at the First Affiliated Hospital of Guangxi Medical University from October 2009 to June 2019, and they were divided into 2 cohorts: no significant fibrosis group (n = 65) and significant fibrosis group (n = 51). Inclusion criteria: based on the diagnostic criteria of the 2015 World Health Organization CHB Guidelines^[2]^: all patients who were hepatitis B surface antigen (HBsAg) positive for more than 6 months and those whose HBsAg and/or HBV DNA was still positive. Not included criteria:

1. patients with other viral infections or autoimmune liver diseases, inherited metabolic liver disease, decompensated cirrhosis, and systemic diseases, such as rheumatism, systemic lupus erythematosus, and diabetes; and
2. patients who received antiviral therapy within 6 months before the liver biopsy.

The patients’ baseline situation and the following laboratory parameters were collected at the time of liver biopsy: age, gender, ALT, aspartate transaminase (AST), AST/ALT, alkaline phosphatase (ALP), gamma-glutamyl transpeptidase (GGT), albumin, prealbumin (PA), globulin, cholinesterase (CHE), total bile acid (TBA), total bilirubin, indirect bilirubin, direct bilirubin, direct bilirubin to total bilirubin ratio (D/T), activated partial thromboplastin time, international normalized ratio, fibrinogen, white blood cells, thrombocytocrit, platelet (PLT), HBeAg status, and HBV DNA levels.

### 2.2 Liver biopsy

Liver puncture was performed as a part of their medical evaluation before the initiation of treatment, and the histopathological material pertinent to the current study was taken during that procedure. After the informed consent form was signed by the parents, a liver biopsy was performed on the patients under ultrasound guidance using an automated biopsy ejection 18G/16G cutting needle (BARD Max-Core Disposable Biopsy Instrument, USA). The standard liver tissue confession requires a length of ≥1 cm and a number of portal areas of ≥6. The specimens were fixed in a 10% formaldehyde solution to make conventional paraffin sections, which underwent hematoxylin-eosin and Masson staining. According to the Scheuer system standard, liver fibrosis is classified into 5 stages: S0, no fibrosis; S1, enlarged, fibrotic portal tracts; S2, periportal or portal–portal septa but intact architecture; S3, fibrosis with architectural distortion but no obvious cirrhosis; and S4, probable or definite cirrhosis.^[9]^ Significant fibrosis was defined as fibrosis stage ≥ S2. The specimens were evaluated by 2 independent pathologists.

This study was approved by the ethics committee of the First Affiliated Hospital of Guangxi Medical University, and written informed consent was obtained from all parents or guardians.

### 2.3 Statistical analysis

IBM SPSS 22.0 and MedCalc 19.07 were used for data analysis. The Kolmogorov–Smirnov test was used for the normality analysis of the variables. The data are represented by the median (25th and 75th percentiles). The Spearman correlation coefficient was used to analyze the relationship between the variable parameters and liver fibrosis stages. An independent samples *t*-test (normal distribution data) and the Mann–Whitney *U* test (non-normal distribution data) were used to compare the differences in the variables between the 2 groups (no significant fibrosis group and significant fibrosis group). The logarithm of the variables that were statistically significant in the difference and correlation analyses were used in the logistic regression analysis to establish the noninvasive model. The receiver operating characteristic (ROC) curve analysis was used to evaluate the diagnostic accuracy of the model and to select the optimal cut-off values. Finally, the area under the ROC (AUROC) of the new model was compared with 2 pre-existing noninvasive indexes (APRI = [AST(IU/L)/ULN] × 100/PLT (×10^9^/L) and FIB-4 = [Age(Y) × AST(IU/L)]/[PLT (×10^9^/L) × ALT (IU/L)^1/2^]) using the DeLong test. *P* < .05 was considered statistically significant.

## 3 Results

### 3.1 General information

A total of 116 patients (80 males, 36 females) were included in the study. The median age was 6 years old. There were 65 (56.03%) cases in the no significant fibrosis group and 51 (43.97%) cases in the significant fibrosis group (Table 1).

**Table 1:** Characteristics of the patients in the training set.

Variable	Training set (n = 116)
Age (y)	6 (3–10)
Male gender (n, %)	80 (68.96)
Liver fibrosis stage^†^ (n, %)	
S0	34 (29.31)
S1	31 (26.72)
S2	32 (27.59)
S3	11 (9.48)
S4	8 (6.90)

† Liver fibrosis stage: the fibrosis score of liver biopsy is taken as the gold standard. S0, no fibrosis; S1, enlarged, fibrotic portal tracts; S2, periportal or portal–portal septa but intact architecture; S3, fibrosis with architectural distortion but no obvious cirrhosis; S4, probable or definite cirrhosis.^[9]^.

### 3.2 Correlation analysis between the variables and liver fibrosis stages

Spearman correlation analysis was used to test the correlation between the variables and the liver fibrosis stages (Table 2). The results showed that TBA, GGT, AST, D/T, and ALT were positively correlated with fibrosis stage and that PA, CHE were negatively correlated with fibrosis stage. All the correlation coefficients of these variables were less than 0.05. It was difficult to assess liver fibrosis using a single indicator in children with CHB.

**Table 2:** Spearman's correlation between the variables and the liver fibrosis stage.

Variable^†^	Training set (n = 116)	*R* value	*P* value
Age (yr)	6.00 (3.00–10.00)	–0.129	.167
D/T	0.30 (0.30–0.40)	0.203	.029^∗^
ALT (U/L)	55.00 (33.00–150.00)	0.187	.045^∗^
AST (U/L)	57.50 (35.50–127.00)	0.286	.002^∗∗^
AST/ALT	1.00 (0.70–1.30)	0.095	.312
GGT (U/L)	19.00 (15.00–39.65)	0.329	.000^∗∗^
ALP (U/L)	249.00 (220.00–296.00)	–0.002	.980
Globulin (g/L)	25.30 (22.70–28.70)	0.045	.635
Albumin (g/L)	43.40 (41.50–45.30)	0.061	.514
Prealbumin (mg/L)	163.90 (141.85–190.45)	–0.285	.002^∗∗^
Cholinesterase (U/L)	8194.00 (7348.50–9567.00)	–0.191	.004^∗∗^
Total protein (g/L)	68.55 (64.65–71.90)	0.034	.713
Total bile acid (μmol/L)	10.30 (5.85–20.80)	0.292	.001^∗∗^
Total bilirubin (μmol/L)	6.30 (4.80–10.30)	0.041	.663
Indirect bilirubin (μmol/L)	4.00 (3.05–6.65)	–0.040	.669
Direct bilirubin (μmol/L)	2.20 (1.50–3.20)	0.138	.139
APTT (s)	35.00 (32.55–37.45)	0.156	.094
INR (s)	0.95 (0.91–1.00)	–0.039	.678
Thrombin time (s)	11.60 (10.90–12.40)	–0.051	.584
Fibrinogen (g/L)	2.83 (2.59–3.28)	–0.165	.077
Prothrombin time (s)	11.20 (10.60–11.80)	0.007	.942
MPV (fl)	8.19 (7.64–9.21)	0.055	.555
Platelet count (10^9^/L)	287.90 (245.00–336.10)	–0.113	.227
White blood cell (10^9^/L)	7.67 (6.10–9.66)	–0.040	.669
Thrombocytocrit (ml/L)	0.24 (0.20–0.28)	–0.078	.405
Lg [HBVDNA (copies/ml)]	7.36 (6.72–8.01)	–0.054	.567

† Variables are presented as the median (interquartile range [IQR]). ALP = alkaline phosphatase, ALT = alanine transaminase, APTT = activated partial thromboplastin time, AST = aspartate transaminase, AST/ALT = aspartate transaminase to alanine transaminase ratio, D/T = direct bilirubin to total bilirubin ratio, GGT = gamma-glutamyl transpeptidase, INR = international normalized ratio, Lg = logarithm base 10, MPV = mean platelet volume, R value = correlation coefficient.

### 3.3 Difference analysis between the no significant fibrosis group and the significant fibrosis group

According to liver biopsy, the patients were divided into 2 groups: no significant fibrosis group (S <2, n = 65) and significant fibrosis group (S ≥2, n = 51). Table 3 shows the difference analysis between the 2 groups. The results suggested that PA (*P* = .002), ALT (*P* = .045), AST (*P* = .002), CHE (*P* = .004), GGT (*P* = .000), and TBA (*P* = .001) were independent predictors of significant fibrosis.

**Table 3:** Difference analysis between the 2 groups in the training set.

Variable^†^	No significant fibrosis n = 65	Significant fibrosis n = 51	Statistical value	*P* value
Age (yr)	3.00 (6.00–10.00)	2.00 (6.00–9.50)	*Z* = –0.648^§^	.517
D/T	0.30 (0.30–0.40)	0.40 (0.30–0.40)	*Z* = –1.639^§^	.101
ALT (U/L)	45.00 (28.00–119.00)	72.00 (39.50–203.00)	*Z* = –2.128^§^	.033^∗^
AST (U/L)	45.00 (30.00–79.00)	76.00 (42.50–173.00)	*Z* = –3.010^§^	.003^∗∗^
AST/ALT	1.00 (0.70–1.30)	1.00 (0.75–1.44)	*Z* = –0.773^§^	.440
GGT (U/L)	17.00 (14.00–21.00)	29.00 (17.00–47.50)	*Z* = –3.873^§^	.000^∗∗^
ALP (U/L)	243.00 (223.00–295.00)	252 (208–295)	*Z* = –0.117^§^	.907
Globulin (g/L)	24.90 (22.40–28.70)	26.00 (23.30–28.70)	*T* = –1.172^‡^	.244
Albumin (g/L)	43.40 (41.50–45.30)	43.40 (41.60–45.35)	*T* = –0.031^‡^	.975
Prealbumin (mg/L)	175.00 (146.60–202.10)	152.60 (126.95–178.70)	*T* = 3.338^‡^	.001^∗∗^
Cholinesterase (U/L)	8691.00 (7519.00–9845.00)	7981.00 (7044.00–8632.50)	*Z* = –2.172^§^	.030^∗^
Total protein (g/L)	68.30 (64.80–71.90)	69.10 (64.70–71.70)	*T* = –0.611^‡^	.542
Total bile acid (μmol/L)	8.40 (5.10–15.73)	15.50 (6.65–26.00)	*Z* = –2.535^§^	.012^∗^
Total bilirubin (μmol/L)	6.50 (5.00–8.70)	5.70 (4.80–8.70)	*Z* = –0.320^§^	.749
Indirect bilirubin (μmol/L)	4.50 (3.30–6.00)	3.80 (2.90–7.30)	*Z* = –0.281^§^	.779
Direct bilirubin (μmol/L)	2.20 (1.40–2.90)	2.20 (1.60–3.65)	*Z* = –1.303^§^	.193
APTT (s)	34.70 (32.50–37.30)	35.20 (32.90–37.50)	*T* = –0.092^‡^	.926
INR (s)	0.95 (0.91–1.02)	0.94 (0.90–0.99)	*Z* = –0.914^§^	.361
Thrombin time (s)	11.60 (10.90–12.50)	11.60 (10.95–12.20)	*T* = –0.090^‡^	.929
Fibrinogen (g/L)	2.89 (2.62–3.28)	2.68 (2.51–3.27)	*Z* = –1.196^§^	.232
Prothrombin time (s)	11.20 (10.70–12.00)	11.10 (10.60–11.70)	*Z* = –0.818^§^	.413
MPV(fl)	8.13 (7.50–8.97)	8.21 (7.92–9.34)	*Z* = –1.179^§^	.238
Platelet count (10^9^/L)	288.70 (250.80–343.20)	282.60 (240.70–330.00)	T = 0.763^‡^	.447
White blood cell (10^9^/L)	7.64 (6.25–10.13)	8.05 (5.80–9.35)	*Z* = –1.093^§^	.274
Thrombocytocrit (ml/L)	0.25 (0.20–0.29)	0.24 (0.21–0.28)	*T* = –0.001^‡^	.900
Lg[HBVDNA(copies/ml)]	7.39 (6.73–8.28)	7.32 (6.77–7.81)	*Z* = –1.046^§^	.296

† Variables are presented as the median (interquartile range [IQR]).

‡ Normal distribution data: Independent sample t-test.

§ Non-normal distribution data: Mann–Whitney *U* test. ALP = alkaline phosphatase, ALT = alanine transaminase, APTT = activated partial thromboplastin time, AST = aspartate transaminase, AST/ALT = aspartate transaminase to alanine transaminase ratio, D/T = direct bilirubin to total bilirubin ratio, GGT = gamma-glutamyl transpeptidase, INR = international normalized ratio, Lg = logarithm base 10, MPV = mean platelet volume.

### 3.4 Establishment of the formula for the noninvasive model and the selection of cut-off values

After taking the logarithmic change in the variables with statistical significance in the difference analyses and Spearman correlation analyses, the fit of the multiple models was calculated using logistic regression analysis. The final model was determined using the backward stepwise procedure: Y = –1.803LN (PA (mg/L)) + 0.769LN (GGT (U/L)) + 6.436 (AUROC 0.732, 95% confidence interval [CI] 0.642–0.810). As the formula was difficult to calculate in the application, we used mathematical relations to simplify it and obtained the G index (AUROC 0.733, 95% CI 0.643–0.811): G Index = 600 × GGT(U/L)/(PA(mg/L))^2^.

A G index value ≤0.28 could be considered no significant liver fibrosis was found (sensitivity 86.27%, specificity 38.46%). Among the 51 patients in the significant fibrosis group, only 7 cases (13.73%; 6 cases in S2 and 1 case in S3) had a G index value ≤0.28. In all 116 patients, the negative predictive value (NPV) was 78.13%, and the diagnostic accuracy (DA) was 59.48% (69/116) (Table 4).

**Table 4:** Accuracy of the G index and APRI in predicting significant fibrosis.

Model	Patients n = 116 n (%)	S0–1^†^ n = 65 n (%)	S2–4^†^ n = 51 n (%)	Sensitivity (%)	Specificity (%)	PPV (%)	NPV (%)	DA (%)
G index								
≤0.28	32 (28)	25 (38)	7 (14)	86.27	38.46	52.38	78.13	59.48
>0.28	84 (72)	40 (62)	44 (86)					
≤1.16	88 (76)	57 (88)	31 (61)					
>1.16	28 (24)	8 (12)	20 (39)	39.22	87.69	71.43	64.77	66.38
APRI								
≤ 0.26	28 (24)	21 (32)	7 (14)	86.27	32.31	50.00	75.00	56.03
>0.26	88 (76)	44 (68)	44 (86)					
≤0.9	90 (78)	57 (88)	33 (65)					
>0.9	26 (22)	8 (12)	18 (35)	35.29	87.69	69.23	63.33	64.66
FIB-4								
≤0.05	14 (12)	9 (14)	5 (10)	88.24	12.31	45.10	64.29	47.41
>0.05	102 (88)	56 (86)	46 (90)					
≤0.4	104 (90)	60 (92)	44 (86)	17.65	95.38	58.33	57.69	57.76
>0.4	12 (10)	5 (8)	7 (14)					

† According to the Scheuer system standard, liver fibrosis is classified into 5 stages: S0, no fibrosis; S1, enlarged, fibrotic portal tracts; S2, periportal or portal–portal septa but intact architecture; S3, fibrosis with architectural distortion but no obvious cirrhosis; and S4, probable or definite cirrhosis.^[9]^. S0–S1 mean without significant liver fibrosis; S2–S4 mean significant liver fibrosis involvement. DA = diagnostic accuracy, NPV = negative predictive value, PPV = positive predictive value.

A G index value > 1.16 could be considered that significant liver fibrosis was found (specificity 87.69%, sensitivity 39.22%). This specificity suggests that the majority of children without significant liver fibrosis had a G index value ≤1.16. Among the 65 children with CHB without significant liver fibrosis, only 8 (12.31%) had a G index >1.16, and the positive predictive value (PPV) was 71.43%. Among the 116 children with CHB, 20 of the 28 children with G index >1.16 had significant liver fibrosis. The DA was 66.38% (77/116), as shown in Table 4. When the values of the G index were outside 0.28 and 1.16, children with CHB could reduce the need for liver biopsy by up to 52% of individuals, with an overall accuracy of 75%.

### 3.5 Comparison of the G index model with two pre-existing noninvasive index models

**Figure 1:**
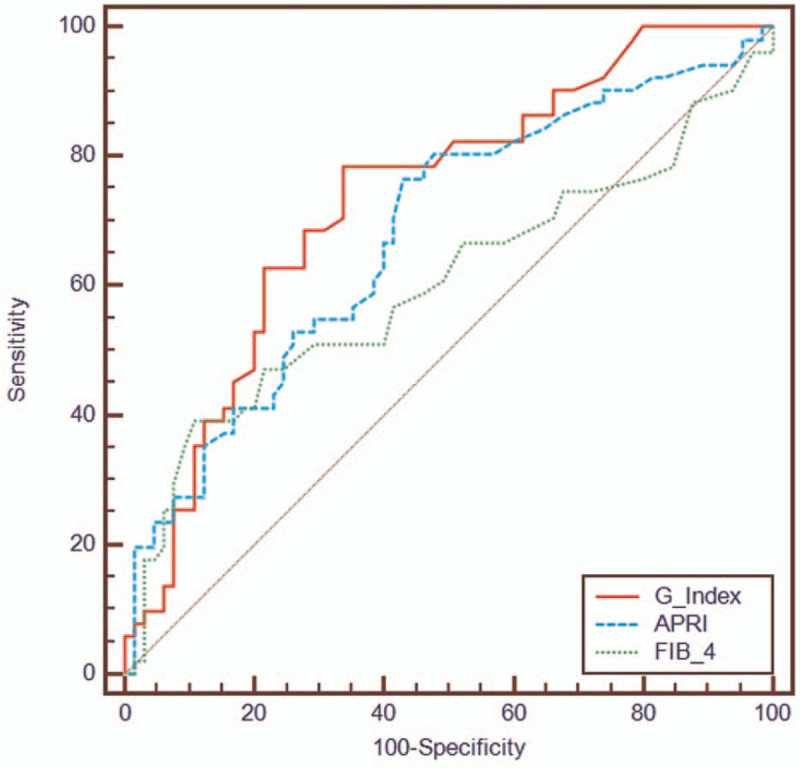
Comparison of the ROC curves of different predictive models in predicting significant fibrosis.

MedCalc 19.07 was used to analyze the AUROC of the models, including APRI, FIB-4, and G Index (Fig. 1). The AUROC of the G Index, APRI, and fibrosis index based on 4 factors (FIB-4) were 0.733 (95% CI 0.643–0.811), 0.680 (95% CI 0.587–0.764), and 0.601 (95% CI 0.506–0.691), respectively. According to the DeLong test, a statistical difference was found between the G index and FIB-4 in children with CHB (*P* = .0365) (Table 5). The optimal cut-off values of the FIB-4 model were 0.05 (sensitivity 88.24%) and 0.40 (specificity 95.38%) to predict significant fibrosis in children with CHB. Using the optimal cut-off values, liver biopsy in 22.41% (26/116) of children with CHB could be avoided. The overall accuracy was 61.54% (16/26), as shown in Table 4. Although there was no significant difference between the G Index and APRI (*P* > .05). The AUROC was <0.7. The optimal cut-off values of the APRI model were 0.26 (sensitivity 86.27%) and 0.90 (specificity 87.69%) to predict significant fibrosis in children with CHB. Using the optimal cut-off values, liver biopsy in 22.41% (26/116) of children with CHB could be avoided. The overall accuracy was 72.22% (39/54), as shown in Table 4.

**Table 5:** Pairwise comparison of the ROC curves.

Model comparison	Difference between areas	Standard error	*P* value	95% Confidence interval
G Index and APRI	0.733–0.680 = 0.053	0.0329	.1061	–0.0113 to 0.118
G Index and FIB-4	0.733–0.601 = 0.132	0.0633	.0365^∗^	0.00830 to 0.256
APRI and FIB-4	0.680–0.601 = 0.079	0.0626	.2068	–0.0437 to 0.202

APRI = aminotransferase-to-platelet ratio index, FIB-4 = fibrosis index based on four factors.

## 4 Discussion

Ikenaga et al^[10]^ found that cirrhosis was reversible. Early intervention could delay the occurrence of cirrhosis and even reduce the incidence of cirrhosis and liver cancer. It was reported that children with CHB who received antiviral treatment for 5 years old could achieve a higher HBsAg clearance rate.^[11]^ Many studies have shown that WFA-positive Mac-2-binding protein,^[12]^ golgi protein 73,^[13]^ and other new molecular biological indicators are independent predictors of significant fibrosis in CHB. However, most studies on new molecular biological indicators are limited to laboratory studies or require validation through a large-scale multicenter trial.^[14,15]^ Currently, most grassroots hospitals cannot provide testing services for these new molecular indicators. The possibility of their widespread use is unclear. Research on non-invasive models of CHB has shown that some information on liver fibrosis can be obtained from routine laboratory results.^[16,17]^ Our study tried to build a simple, non-invasive diagnosis model to reduce the need for liver biopsy in children with CHB.

In this study, the Spearman correlation analysis showed that D/T, TBA, GGT, AST, and ALT were positively correlated with fibrosis stage and that CHE and PA were negatively correlated with fibrosis stage. However, the *P* values of D/T, AST, ALT, and CHE (likelihood ratio test) in the logistic correlation analysis were greater than 0.05, and they did not meet the criteria for variable selection in the logistic regression model.^[18]^ The possible reasons are as follows:

1. There are multiple linear relationships between these variables.
2. The sample size of this study was small.

Therefore, the relationship between these variables and the liver fibrosis stage in children with CHB was not analyzed.

GGT, the variable in the G index model, mainly exists in the cytoplasm of hepatocytes and the epithelium of the intrahepatic bile duct, and it regulates the metabolism of extracellular glutathione.^[19]^ With the development of liver fibrosis, the destruction of hepatocytes increases, and GGT in cells is released into the blood, leading to an increased concentration of GGT in the blood. GGT has been proven to be an independent predictor of liver fibrosis in noninvasive models for adults,^[20,21]^ consistent with the results of our study. PA is an acute reactive protein secreted by hepatocytes and is involved in the transport of vitamin A in vivo. It has a short half-life and can sensitively and accurately reflect the synthetic and metabolic functions of the liver and its nutritional status.^[22]^ When fibrosis progresses, the synthesis and the release of prealbumin decrease. However, confirming the mechanisms of GGT and PA in the progression of liver fibrosis requires more studies.

The diagnostic value of classical adult models (ARPI and FIB-4) for children with CHB was low in our study. The cut-off values of APRI in children were 0.26 and 0.90, which were different from adults (0.5 and 1.5).^[7]^ When using our cut-off values, APRI could reduce 47% of patients’ need for liver biopsy. The AUROC of FIB-4 was significantly lower than that of the G index (*P* < .05). Moreover, age is included in the FIB-4 formula. We speculated that the cut-off values of children were different from those of adults, which was confirmed in the study. The cut-off values of FIB-4 in children were 0.05 and 0.4, which were significantly different from those in adults (1.45 and 3.25).^[8]^ Thus, APRI and FIB-4 are not completely suitable for children with CHB.

This study has some limitations. This work is a retrospective analysis, and the G index model needs more cases for confirmation. Currently, the ROC curve analysis is generally used to evaluate diagnostic efficacy. However, this analysis method is easily affected by the uneven distribution of disease degree. Some scholars have proposed the use of the DANA formula to correct the effect of the incidence of fibrosis stages.^[23]^ But this public formula was obtained from the analysis of patients with chronic hepatitis C, and it is uncertain whether the DANA formula is also applicable to patients with CHB.

## 5 Conclusion

Classical noninvasive models for adults (FIB-4 and APRI) cannot be completely applied to children with CHB. The G index, which is made up of GGT and PA, is a simple model in clinical practice. As it can predict and exclude significant fibrosis in children with HBV, it may reduce the need for liver biopsy in children with CHB.

## Author contributions

**Data curation:** Kang-Ling Zhang, Qing Tang.

**Formal analysis:** Xiu-Qi Chen.

**Methodology:** Kang-Ling Zhang, Xiu-Qi Che, Zi-Li Lv.

**Project administration:** Qing Tang.

**Resources:** Kang-Ling Zhang.

**Software:** Xiu-Qi Chen.

**Supervision:** Zi-Li Lv.

**Validation:** Qing Tang.

**Visualization:** Qing Tang.

**Writing – original draft:** Kang-Ling Zhang.

**Writing – review & editing:** Xiu-Qi Chen, Qing-wen Shan.
